# Why do families still not receive the child support grant in South Africa? A longitudinal analysis of a cohort of families across South Africa

**DOI:** 10.1186/1472-698X-12-24

**Published:** 2012-10-22

**Authors:** Wanga Zembe-Mkabile, Tanya Doherty, David Sanders, Debra Jackson, Mickey Chopra, Sonja Swanevelder, Carl Lombard, Rebecca Surender

**Affiliations:** 1 Medical Research Council, Francie van Zyl Drive, Parow, Cape Town, South Africa; 2 School of Public Health, University of the Western Cape, Modderdam Road, Belville, South Africa; 3 UNICEF, UNICEF House, 3 United Nations Plaza, New York, 10017, NY, USA; 4 Department of Social Policy and Intervention, Oxford University, Oxford, UK

## Abstract

**Background:**

Child cash transfers are increasingly recognised for their potential to reduce poverty and improve health outcomes. South Africa‘s child support grant (CSG) constitutes the largest cash transfer in the continent. No studies have been conducted to look at factors associated with successful receipt of the CSG. This paper reports findings on factors associated with CSG receipt in three settings in South Africa (Paarl in the Western Cape Province, and Umlazi and Rietvlei in KwaZulu-Natal).

**Methods:**

This study used longitudinal data from a community-based cluster-randomized trial (PROMISE EBF) promoting exclusive breastfeeding by peer-counsellors in South Africa (ClinicalTrials.gov: NCT00397150). 1148 mother-infant pairs were enrolled in the study and data on the CSG were collected at infant age 6, 12, 24 weeks and 18–24 months. A stratified cox proportional hazards regression model was fitted to the data to investigate factors associated with CSG receipt.

**Results:**

Uptake of the CSG amongst eligible children at a median age of 22 months was 62% in Paarl, 64% in Rietvlei and 60% in Umlazi. Possessing a birth certificate was found to be the strongest predictor of CSG receipt (HR 3.1, 95% CI: 2.4 -4.1). Other factors also found to be independently associated with CSG receipt were an HIV-positive mother (HR 1.2, 95% CI: 1.0-1.4) and a household income below R1100 (HR1.7, 95% CI: 1.1 -2.6).

**Conclusion:**

Receipt of the CSG was sub optimal amongst eligible children showing administrative requirements such as possessing a birth certificate to be a serious barrier to access. In the spirit of promoting and protecting children’s rights, more efforts are needed to improve and ease access to this cash transfer program.

## Background

Cash transfer programs have increasingly become the strategy of choice for poverty alleviation in middle and low-income regions such as Latin America and Caribbean countries (LAC), Asia, and Africa [1,2]. Many of these programs have been found to be effective in improving child outcomes such as learning outcomes, child growth and nutrition. However, several of the child cash transfer programs that have been evaluated and rolled out at scale are conditional cash transfers (CCTs). Conditionality refers to post-grant conditions that require recipients to change their behaviour for purposes of human capital development. Very few developing countries have unconditional child cash transfer programs although they are common in developed countries such as the United Kingdom and several other European Union countries. In Africa, there are a number of unconditional cash transfer programs that exist in countries such as Zambia, Malawi, Ghana, and Kenya but these are either small scale (often pilot programs covering less than 100 000 beneficiaries) or they are not exclusively targeted at children [3].

Debates on conditional and unconditional cash transfers centre on two main contending arguments. Proponents of conditional cash transfers argue that conditioning transfers on behavioural change helps to address not only immediate poverty but long-term poverty through its insistence on human capital investment [4]. However, advocates of unconditional cash transfers believe that human capital investment in beneficiaries can be enhanced without enforcing conditionality. This group assert that conditionality is a violation of social protection as a human right since it interferes with beneficiaries’ right to choose on what and how the transfer should be spent [5].

CCTs which have garnered world-wide praise and great interest are from the LAC countries, specifically *Bolsa Familia* in Brazil and Opportunidades in Mexico (formerly called *Progresa*). Both programs are targeted and non-contributory, focusing on the health, education and nutrition of children from the poorest households in the two countries [4,6-8]. The two CCTs have been associated with high rates of school enrolment and attendance, improved child growth (lower prevalence of stunting), increased utilisation of health care services, and in the case of *Bolsa Familia*, contributing to a reduction in inequality [7,8].

South Africa is unique among developing countries for establishing a large scale cash transfer program, the Child Support Grant (CSG), for children of poor families. While the CSG is not conditional on behavioural change it does have requirements, which need to be met by prospective recipients. These are: possession of a birth certificate for the child, an identity document for the mother, hospital card, Road to Health Card (infant health record), and a parental income that is currently set at less than R2800 (US$340) per month for single parents, and R5600 (US$680) for married couples. At the time of the study eligibility was below R1100 parental income per month for urban areas and below R800 for rural areas. Recently other requirements for school enrolment and attendance for children between the ages of 7 and 18 years have been added. This new amendment to CSG legislation emphasises that proof of school enrolment and attendance is not a condition for continued receipt of the CSG, but is there to help identify and help children who are struggling to remain in the school system [9]. The CSG was introduced 14 years ago as a response to childhood poverty and it constitutes the largest cash transfer in the continent, both in terms of coverage ( over 10 million children) and the state budget allocated to it (US$695 million per annum) [10]. Over the years, the age limit for eligibility has been repeatedly extended, most recently to 18 years of age. The target set by the Department of Social Development is to reach 100% of all eligible children in the country.

During the early stages of its implementation the CSG was plagued with roll out challenges such as low uptake rates, which have since improved [11]. More recently there have been other debates around conditionality and targeting, with some advocating for continued receipt of the grant to be tied to conditions, and others arguing for universal access instead of means testing for eligibility [12,13]. The discourse on conditionality in South Africa has been spurred in part by the success of conditional cash transfer programs in LAC countries.

There has been a general paucity of empirical evidence on the performance of the CSG. The few studies that have been conducted have looked at the grant’s reach, utilisation and impact. A national survey conducted by Delany and colleagues in 2008 with some 2700 households in South Africa showed that the CSG is reaching 80% of its intended beneficiaries among all eligible children 0–14 years, constitutes 40% of the household income in poor families, and that 80% of it is used for food, clothing and school related costs [14]. Additionally, more recent evidence shows that take up rates of the CSG are highest between the ages of 7 to 10 years and are lower for infants and adolescents, and that early receipt of the grant is associated with improved child height for age for children whose mothers have higher education levels; and more grades in school for girl children whose mothers have lower education attainment, as well as reduced risky behaviours amongst adolescents [15]. Another study showed that the CSG is positively correlated with improved child nutritional status, and that early receipt of the grant in the first few months of life is critical to achieving this outcome [16]. These studies validate the importance of CSG access and timing of receipt in maximising the benefits of the grant for each child. The study by Delany and colleagues while showing that receipt of the CSG may be high at a national level, conversely highlights the 20% of eligible children who are not receiving the grant, and hides the variations in take up rates present at the provincial level and within age categories [17,18].

In the absence of strong evidence on the effectiveness of unconditional cash transfers on poverty alleviation and human capital development, there has been a significant move towards replicating the LAC model of conditional cash transfer programs in developing countries all over the world, including Sub-Saharan Africa [2,13]. The CSG thus presents a unique opportunity to further our understanding of how an unconditional cash transfer works in a developing country context. This paper presents findings on the factors associated with receipt of the unconditional CSG across three diverse settings in South Africa.

## Methods

### Study design

This study used data from a community-based cluster-randomized trial promoting exclusive breastfeeding by peer-counsellors in three South African sites between 2006 and 2008. A total of 34 clusters from three separate areas in South Africa were chosen: Paarl in the Western Cape Province (peri-urban), Umlazi (urban) and Rietvlei (rural) in KwaZulu-Natal Province. Infant mortality rate (IMR) and antenatal HIV prevalence at the time of the study were 40/1000 and 10% in Paarl, 60/1000 and 42% in Umlazi and 99/1000 and 34% in Rietvlei. Full trial study methods are described elsewhere [19]. In the intervention arm women received 5 visits from peer supporters to promote exclusive breastfeeding and in the control arm women received the same number of visits from peer supporters but they were counselled on how to apply for the child support grant. The visits took place antenatally, and at 1 week, 4 weeks, 7 weeks and 10 weeks after birth. The trial found no effect of peer support visits on uptake of the child support grant at six months of age. Results of CSG receipt by arm were 54% in the infant feeding arm and 46% in CSG arm, but these results were not significant (Relative risk 1.0, 95% CI: 0.9 -1.2), therefore this paper analysed the study as a longitudinal cohort adjusting for study arm and clustering.

A total of 1276 mother-infant pairs were recruited. Among these, 128 were excluded due to relocation or being lost-to-follow-up, twin delivery, death of infant or mother before 3 weeks after birth. Thus, 1148 mother-infant pairs remained in the analysis. The mother-infant pairs were scheduled to be interviewed at recruitment (antenatally) for socio-demographic information and at 3, 6, 12 and 24 weeks after birth for data regarding CSG uptake, with a final follow up visit amongst a sub sample of 741 children at a median age of 22 months (range 9–36 months) to assess final grant receipt. Detailed information on CSG application and receipt was only collected at the final follow-up interview to allow enough time for families to have gone through the grant process. Contact was made with mothers for the final follow up visit through home visits by data collectors. Where possible, mothers were first called on their cell phones to determine a suitable time for the visit.

### Data collection and management

A structured paper based questionnaire was administered at the recruitment, 3, 6, 12 and 24 week data collection points by a trained data collector. Items in the questionnaire included socio demographics, infant feeding practices, grant application and receipt. At the final follow up interview data collectors captured the data using questionnaires loaded onto cell phones purchased for the study with built in range checks and skip logic. The questionnaires were automatically transferred to a central server once each one was completed. Items in this final questionnaire included timing of CSG application, barriers to CSG access, use of the CSG, anthropometry, and infant health. Data collectors were not involved in the implementation of the intervention. Data collected on paper were double entered using EpiData (http://www.epidata.dk) and merged with the data collected from the final interview on the cell phones.

### Data analysis

CSG receipt was defined as a mother reporting receipt of the grant at any of weeks 12, 24 or the final data collection point (median age 22 months). Possession of a birth certificate was defined as the mother reporting possessing a birth certificate for her child at any of weeks 6, 12 or 24.

A survival analysis approach was used to model time to receipt of a CSG on a set of determinants. A stratified Cox proportional hazards model was fitted and hazard ratio estimates were obtained, with equal coefficients across strata (sites) but with a baseline hazard unique to each strata. The Breslow method was used for handling tied successes due to the limited number of data collection points. An epidemiological approach was undertaken in selecting variables for the model including demographic and socio-economic factors relevant to the South African context. In case of collinearity, one of the variables, such as Identity Document (ID) was collinear with Birth Certificate, and was subsequently dropped from the model. The models were adjusted for arm and clustering to account for the community randomised trial design. Possible interaction terms which included maternal education and arm; maternal education and birth certificate; birth certificate and arm were inserted into the model but none were found significant to include in the model.

A socioeconomic wealth index was constructed with the use of multiple correspondence analysis based on ownership of assets including mobile phone and television, and house characteristics including water source, roof material and toilet type. This method is analogous to principal component analysis, and better suited for categorical data [20]. The infants’ households were grouped into quintiles on the basis of socioeconomic rank. Data analysis was done with SAS version 9.2 and STATA/IC 12.0.

### Ethics

Ethics approval for the cluster randomised controlled trial was received from the Ethics Committee of the Medical Research Council South Africa. Signed or thumb-printed informed consent was obtained from each mother prior to study participation. Additional ethics approval was granted in a subsequent application to the Medical Research Council Ethics Committee for the additional data collection point when the children were at median age of 22 months. An information sheet explaining the purpose of the additional interview was read to each participant and each participant who agreed to participate signed a consent form. This study is registered with ClinicalTrials.gov, number NCT00397150.

## Results

### Characteristics of mothers

Table 1 shows the demographics of the participants in the study by site. The mean age of mothers was similar in Paarl and Rietlvei (24 years) and slightly younger in Umlazi at 23 years. Educational levels of mothers were lower in Rietvlei with a median of 9 years schooling compared to 10 years in Paarl and 11 in Umlazi. Incomes also differed across the three sites, with a median of R1010 household income per month in Paarl, R1000 in Umlazi, and notably lower in Rietlvei at R780 per month. Most of the mothers in Paarl were single (75%), as was the case in Umlazi (67%), with Rietvlei having the highest proportion of married mothers (57%).

**Table 1:** Mothers’ characteristics by site

**Characteristics**	**Paarl (n = 351)**	**Rietvlei (n = 308)**	**Umlazi (n = 489)**
**Mother’s Age**			
Mean years (SD)	24.9 (6.3)	23.9 (6.1)	24 (5.6)
**Mother’s education level**			
Median years (Q1-Q3)	10 (9–12)	9 (8–11)	11 (10–12)
**Marital status**			
Single	262 (74.6)	126 (41.5)	326 (66.7)
Married	62 (17.7)	172 (56.6)	34 (6.9)
Cohabiting	27 (7.7)	6 (1.9)	129 (26.4)
**SES quintile**			
1 (poorest)	0	183 (61.8)	12 (3.0)
2	35 (10.5)	84 (28.3)	68 (17.3)
3	82 (24.7)	26 (8.7)	95 (24.2)
4	120 (36.2)	3(1.0)	101 (25.7)
5 (least poor)	94 (28.4)	0	116 (29.5)
**Mothers HIV status**			
Positive	21 (5.9)	25(8.1)	138(28.2)
**Household size**			
Mean number of members	5 (4–7)	6 (4–8)	7 (5–10)

Data are number (%) or mean (Std Dev) or median (IQR).

Across the three sites socio-economic status varied widely. Rietvlei had the highest proportion (62%) of participants who were in the poorest quintile compared to Paarl where there were no participants who fell within that quintile. Umlazi had the highest proportion of participants who were in the least poor quintile (30%), Paarl had 28%, and Rietlvei had none. Umlazi had the most HIV positive mothers (28%) and Paarl had the least (6%), with Rietvlei at 8%.

### Indicators of CSG application and receipt

Table 2 shows basic indicators of CSG application and receipt. The majority of mothers applied for the CSG when their children were older than three months of age. Thirty-four percent of the mothers applied for the CSG more than 6 months after their child’s birth, and 26% of the mothers applied for it more than 3 months after their child’s birth. Rietvlei had the highest proportion of mothers who applied for the grant 6 months after their child’s birth (44%), compared to Paarl (35%) and Umlazi (26%). Distances to social services and home affairs offices for application varied from site to site with Rietvlei being the furthest since it is a rural area, however in all cases applicants had to take a bus or taxi (minibus) to get to the nearest office.

**Table 2:** Indicators of CSG application and receipt by site

	**Paarl**	**Rietvlei**	**Umlazi**	**Total**
**Detailed CSG Indicators from the follow-up sample**				
Child age at application (n=549/741)*				
<1 month	17 (9.3)	7 (4.5)	34 (16.0)	58 (10.5)
1 to < 3 months	63 (34.6)	38 (24.5)	60 (28.3)	161 (29.3)
3 to < 6months	38 (20.8)	42 (27.1)	62 (29.2)	142 (25.8)
>= 6 months	64 (35.1)	68 (43.8)	56 (26.4)	188 (34.2)
Waiting longer than a week for a response to the application (n=548/741)*	179 (98.3)	66 (42.8)	9 (4.2)	254 (46.3)
Reasons for CSG application more than 3 months after birth (491/741)*				
Long waiting list	115 (68.0)	99 (65.1)	29 (11.7)	243 (49.4)
Identity document	17 (10.0)	14 (9.2)	35 (20.5)	66 (13.4)
Birth certificate	10 (5.9)	37 (24.3)	29 (17.0)	76 (15.4)
Road to health card	3 (1.7)	1 (0.6)	17 (10.0)	21(4.2)
No Proof of Income	4 (2.3)	0	3 (1.7)	7(1.4)
Misinformation about eligibility criteria	1 (0.6)	0	1 (0.5)	2 (0.4)
Mother lazy	7 (4.1)	0	0	7(1.4)
Proof of residence	0	0	2 (1.1)	2(0.4)
Baby too young	1 (0.6)	0	1 (0.5)	2(0.4)
Underage mother	0	0	2 (1.1)	2(0.4)
No reason	10 (4.1)	0	45 (26.4)	55(11.2)
Reason unclear	1 (0.6)	1 (0.6)	6 (3.5)	8(1.6)
Median age at CSG receipt in months	6 (4-9)	7 (5-10)	5 (3-8)	6 (4-9)
Reasons for CSG non receipt (n= 196)**				
Waiting for response from welfare office	10(9.8)	7 (14.2)	2 (8.8)	19 (9.6)
No ID	13 (12.7)	20 (40.8)	17 (37.7)	50 (25.5)
No birth certificate	2 (1.9)	6 (12.2)	4 (8.8)	12 (6.1)
No Road to Health Card	1 (0.9)	1 (2.0)	0	2 (1.0)
Proof of income	7 (6.8)	0	0	7 (3.5)
Parental income above threshold	31 (30.3)	10 (20.4)	13 (28.8)	54 (27.5)
No time to go to welfare office	19 (18.6)	0	1 (2.2)	20 (10.2)
Problems with documents	3 (2.9)	0	6 (13.3)	8 (4.0)
No money for transport	0	1 (2.0)	0	1 (0.5)
Misinformation about eligibility	2 (1.9)	1 (2.0)	0	3 (1.5)
No reason	11 (10.7)	1 (2.0)	0	12 (6.1)
In receipt of another grant for the same child	0	2 (4.0)	0	2 (1.0)
Reason unclear	3 (2.9)	0	0	3 (1.5)
**CSG eligibility and uptake at end of follow up**				
		Paarl	Rietvlei	Umlazi
CSG eligibility by site		171/316	171/304	225/463
		(54.1) §§	(56. 3)§	(48.6)§§
CSG receipt by median age of 22 months amongst eligible children		116/186 (62. 4)§§	110/171 (64.3)§	168/279 (60.2)§§

Data are number (%) or median (IQR).

Total n differs for the outcomes here as there are different denominators; *women who applied for the grant out of 741 follow-up sample; **women who never received the grant.

Eligibility criteria differs by site as there were different criteria for rural and urban areas at the time of the study; § rural income threshold (<R800 per month) applicable to Rietvlei, §§ urban income threshold (<R1100) applicable to Paarl and Umlazi.

The reasons for taking more than 3 months to apply for the CSG included long waiting lists at Department of Social Development (49%), not having a birth certificate (15%), the mother not having an ID document (13%), and 11% who said they knew of no reason at all. At site level Rietvlei had a higher proportion (24%) of children who received the CSG more than 3 months after birth because of not possessing a birth certificate compared to Paarl (6%) and Umlazi (17%). The median age at grant receipt ranged from 5 months in Umlazi to 7 months in Rietvlei.

The most cited reasons by mothers for not receiving the CSG at all at the time of final follow-up were not possessing an identity document (26%), and not qualifying for receipt because of perceived high financial status (28%). Other reasons for non-receipt included mothers saying they were still waiting for a response from the social development office where they had made an application (10%), mothers who are not able to find time to go to the social development office to make an initial application because they are working (10%), 6% who had no birth certificate for their child, and 4% who had problems with the documentation needed to make an application (mismatched names, documents lost, documents not filled in correctly).

Eligibility differed across the three sites as there were separate income thresholds applicable to rural and urban areas at the time of the study. As such, using the rural income threshold Rietvlei had 56% of children who were eligible for the CSG, and using the urban threshold Paarl had 54%, and Umlazi had 49% of children who were eligible for the grant. The number of eligible children who received the CSG at a median age of 22 months was similar across the three sites; with 64%, 62% and 60% of children in Rietvlei, Paarl and Umlazi in receipt of the grant respectively.

### Predictors of receiving the CSG

Table 3 shows the univariable and multivariable analysis of predictors of CSG receipt. In the stratified Cox model after adjusting for clustering and study arm, the most important predictors of CSG receipt were the child having a birth certificate, mother being HIV positive, and family income.

**Table 3:** Cox proportional hazards regression model*

**Characteristics**	**Received CSG #/N (%)**	**Unadjusted HR(95% CI)**	**Adjusted HR (95%CI)**
**Child has a birth certificate**			
No	67/678 (9. 9)	1	1
Yes	611/678 (90.1)	3.06 (2.45 - 3.82)	3.14 (2.41 - 4.09)
**Mother HIV Status**			
Negative	565/678(83.3)	1	1
Positive	113/678 (16. 7)	1.19 (1.06 -1.34)	1.19 (1.03-1.37)
**Marital Status**			
Married	145/675 (21.5)	1	
Cohabiting	98/675 (14.5)	0.63 (0.46 -0.85)	
Single	432/675 (64.0)	0.88 (0.74 -1.05)	
**Socio-economic Status**			
Top quintile, least poor	118/603(19. 6)	1	
2^nd^ quintile	101/603 (16.7)	0.98 (0.81 -1.18)	
3^rd^ quintile	104/603 (17.2)	0.82 (0.61 -1.10)	
4^th^ quintile	154/603 (25.5)	1.15 (0.85-1.54)	
Bottom quintile, poorest	126/603(20.9)	0.86 (0.63 -1.18)	
**Mother’s age**			
>35	38/677(5.6)	1	1
25–35	230/677( 34.0)	1.13(0.80 -1.59)	1.14 (0.81-1.16)
16-24	409/677(60.4)	1.29 (0.85 -1.69)	1.28 (0.94-1.74)
**Total monthly family income**			
>R4000	15/636 (2.4)	1	1
R1200 – < R4000	193/636 (30.3)	1.49 (1.00 -2.23)	1.40 (0.89 - 2.19)
0-R1100	428/636 (67.3)	1.79 (1.24 -2.58)	1.67 (1.09 -2.55)
**Educational level**			
Some primary	72/673 (10.7)	1	1
Some high school	402/673 (59.7)	0.96 (0.80 -1.15)	0.93 (0.75-1.15)
Completed high school	199/673 (29. 6)	0.85 (0.67 -1.08)	0.76 (0.58-1.01)
**Study arm**			
Infant feeding arm	367/677 (54.2)	1	1
Grant arm	310/677 (45. 8)	1.06 (0.90-1.25)	1.04 (0.91 -1.19)

*stratified by site and adjusted for clustering to account for the PROMISE-EBF study design.

In the unadjusted model children who had a birth certificate were 3 times more likely to receive the CSG than children who did not have it. The likelihood of a child receiving the grant when possessing a birth certificate changed little in the adjusted model (HR 3.14, 95% CI: 2.41-4.09). The HIV positive status of a mother was a modest predictor of CSG receipt, with HIV positive mothers being 1.19 times more likely to receive it than HIV negative mothers in the adjusted model.

The adjusted model shows that the poor were more likely to receive the CSG than their better off counterparts, as applicants who earned less than R1100 per month were 1. 7 times more likely to receive the CSG than those who earned more than R4000 per month. There was some evidence that more educated mothers who had completed high school were less likely to receive the CSG than mothers with primary school education, though this effect had borderline statistical significance (HR 0.76, 95% CI: 0.58-1.01, p-value 0.06).

## Discussion

To our knowledge this is the first longitudinal study in South Africa to describe factors associated with receipt of the CSG. Across the three sites around 60% of children who were eligible for the CSG received it by a median age of 22 months. While this take up rate is notably lower than national estimates previously reported by other authors [14], it reveals the variations that exist within specific age cohorts. Importantly, Samson et al. using a General Household Survey panel dataset reported similar take up rates (61%) for children < 2 [18]. This is disconcerting as this is the age group where children are more vulnerable to negative health and nutritional outcomes which have been shown to impact on life chances and economic productivity [21].

The income thresholds used to establish CSG eligibility during the study period show that low income thresholds can prevent the poor from accessing a cash transfer program designed to help them. The poorest site in the study, rural Rietvlei had a low eligibility rate of 56% when using the < R800 income threshold applied at the time, and thus a large number of children who were poor but were just above that threshold could not access the grant. It is thus encouraging to see that the CSG income threshold has since been raised substantially and no longer distinguishes between rural and urban inhabitants.

The study indicates that barriers to access for eligible families are still present. The most important barrier faced by families was the lack of a birth certificate for their child. A recent evaluation of the *Bolsa Familia* conditional cash transfer programme in Brazil found lack of a birth certificate to be related to poor anthropometric indicators in children since lack of a birth certificate is seen as an indicator of extreme poverty [22]. Obtaining a birth certificate is the final step in a series of steps which start with the child’s mother needing an identity document, transport and money to apply for the documents. In our study Rietvlei site had the lowest proportion of children with birth certificates and the highest proportion of participants in the poorest socio-economic status quintile highlighting the role that poverty plays in accessing the CSG.

There are several measures that could be taken to improve access to the CSG which include mobile outreach programs and activities, interim grants, and strengthened collaboration between departments. The proposed re engineering of Primary Health Care (PHC) in South Africa which includes the establishment of ward based outreach teams with community health workers, could be used to identify households with newborns early and link them with appropriate service providers that would help with CSG application and receipt. The Community Development Workers (CDWs), present in some provinces could also be resource persons to assist families with grant applications. The CDW program exists within the Department of Public Service and Administration to ensure access to housing, child grants, old-age pensions and other services [23].

Opportunities also exist to utilise outreach efforts to improve collaboration between departments responsible for furnishing documents necessary for CSG application. An example is the use of mobile offices which target rural far-out areas; these could have representatives from both the South Africa Social Security Agency who issue grants, as well as the Department of Home Affairs who issue birth certificates and IDs. Areas such as Paarl, where birth certificates are issued in hospitals where women give birth, illustrate the kind of inter-departmental collaboration that is needed to improve access to the CSG.

To deal with the problem of children failing to access the CSG because of outstanding documents such as birth certificates and IDs, the Department of Social Development could provide interim grants to applicants who provide proof of legitimate delays in obtaining required documents. In one area of Kwazulu-Natal such interim grants are provided (personal communication D Sanders). Interim grants would ensure that the rights of needy, deserving children, whose health and development are otherwise compromised by lack of access to the CSG, are protected.

In addition to the above mentioned supply side problems, the finding that most mothers applied for the grant when their children were older than 3 months indicates that some attention should also be paid to demand side issues including raising awareness about the grant at antenatal clinics and possibly through community radio stations.

This study found higher grant receipt amongst HIV positive mothers than HIV negative mothers. One factor that could explain this finding is that HIV positive mothers are likely to have greater access to health services where they can be advised about applying for social grants both for themselves and their children. Access to the CSG also appears to be appropriately reaching the poorest families in South Africa as receipt was associated with a low monthly income. This shows that the CSG is well targeted, reaching its intended recipients.

Our study had some potential limitations. The sites where this research was undertaken were purposely selected and the infrastructural conditions differed greatly between them hence they are not statistically representative of South Africa. They do, however, reflect the most common settings in South Africa, namely urban, peri-urban and rural areas. Due to the observational research design there may be confounders which we did not measure which could be related to CSG access that are not reported on here. The study also has several strengths. The total study sample was large, it included both HIV positive and HIV negative mothers and data on CSG receipt were rigorously collected at four time points. The survival analysis approach enabled optimal use of all available data.

## Conclusion

This paper has shown that administrative requirements for CSG receipt act as barriers to access for children at a crucial early age. In the spirit of promoting and protecting children’s rights, more efforts are needed to improve and ease access to this cash transfer program which is considered to be the most effective poverty alleviation strategy in South Africa [24].

Finally, the fact that the requirements identified in this paper are strongly associated with access raises questions about how much worse take up issues would get were formal conditions to be attached to CSG receipt. As such this calls for careful thought to be given to the decision to implement further barriers in the form of formal conditionalities.

## Competing interests

The authors declare that they have no competing interests.

## Authors’ contributions

WZ, TD, DS planned and wrote the paper. SS and CL were study statisticians. DJ, MC and RS contributed to manuscript design and content. WZ and TD had particular responsibility for study implementation. WZ was active with data management and analytic content. TD and DJ were principal investigators and planned the study design, administered implementation and worked on analytic content. All authors read and contributed towards the final draft.

## Pre-publication history

The pre-publication history for this paper can be accessed here:

http://www.biomedcentral.com/1472-698X/12/24/prepub
